# Dyslipidaemia as a target for atherosclerotic cardiovascular disease prevention in children with type 1 diabetes: lessons learned from familial hypercholesterolaemia

**DOI:** 10.1007/s00125-023-06041-z

**Published:** 2023-11-30

**Authors:** Willemijn E. Corpeleijn, Wouter J. de Waal, Henk S. Schipper, Albert Wiegman

**Affiliations:** 1 Department of Pediatrics, Amsterdam University Medical Centers, location AMC, Amsterdam, the Netherlands; 2 Amsterdam Cardiovascular Sciences, Amsterdam University Medical Centers, location AMC, Amsterdam, the Netherlands; 3 Amsterdam Gastroenterology Endocrinology Metabolism, Amsterdam University Medical Centers, location AMC, Amsterdam, the Netherlands; 4 Diabetes Centraal, Children’s Diabetic Centre, St Antonius Hospital, Utrecht, the Netherlands; 5 Department of Pediatric Cardiology, Wilhelmina Children’s Hospital, University Medical Centre Utrecht, Utrecht, the Netherlands; 6 Center for Translational Immunology, University Medical Centre Utrecht, Utrecht, the Netherlands

**Keywords:** Atherosclerosis, Children, Cholesterol, CVD, Familial Hypercholesterolaemia, Lipid-lowering, Lipids, Prevention, Review, Statins, Type 1 diabetes

## Abstract

In the last few decades, atherosclerotic cardiovascular disease (ASCVD) risk has decreased dramatically among individuals affected by familial hypercholesterolaemia (FH) as a result of the early initiation of statin treatment in childhood. Contemporaneously important improvements in care for people with diabetes have also been made, such as the prevention of mortality from acute diabetic complications. However, individuals with type 1 diabetes still have a two to eight times higher risk of death than the general population. In the last 20 years, a few landmark studies on excess mortality in people with type 1 diabetes, in particular young adults, have been published. Although these studies were carried out in different populations, all reached the same conclusion: individuals with type 1 diabetes have a pronounced increased risk of ASCVD. In this review, we address the role of lipid abnormalities in the development of ASCVD in type 1 diabetes and FH. Although type 1 diabetes and FH are different diseases, lessons could be learned from the early initiation of statins in children with FH, which may provide a rationale for more stringent control of dyslipidaemia in children with type 1 diabetes.

**Graphical Abstract:**

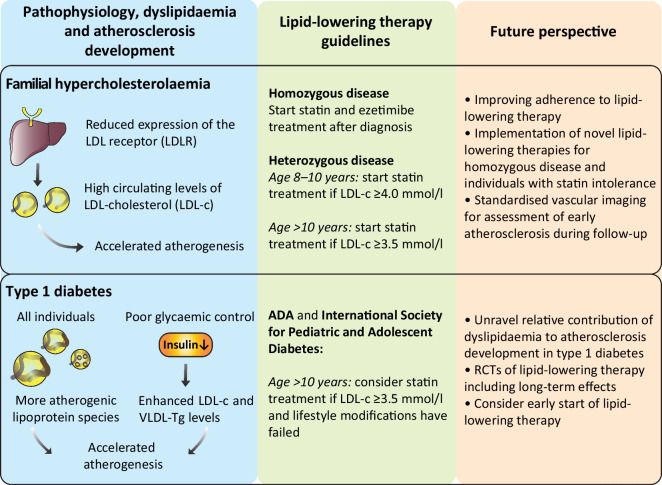

**Supplementary Information:**

The online version contains a slide of the figure for download available at 10.1007/s00125-023-06041-z.


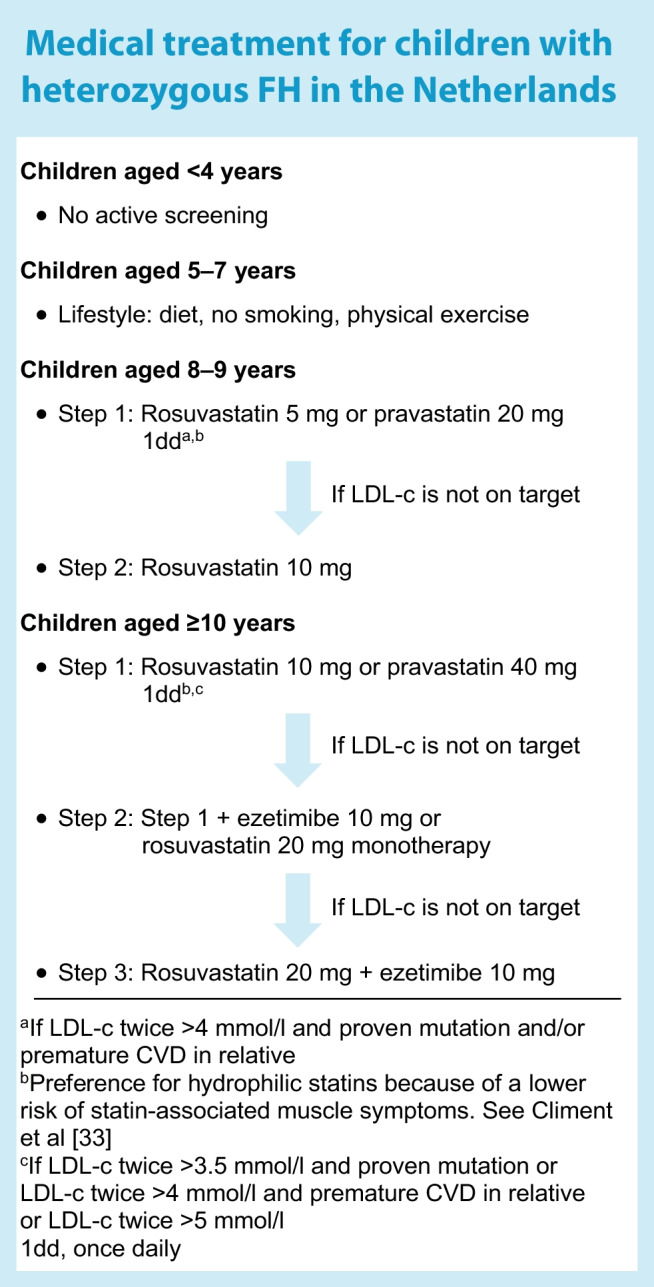


## Cardiovascular disease risk in childhood-onset type 1 diabetes

Mortality rates in individuals with type 1 diabetes increase sharply from age 30 years compared with age-matched control participants without diabetes [1–4]. It appears that the younger an individual is diagnosed with type 1 diabetes, the higher the risk of atherosclerotic cardiovascular disease (ASCVD) [4, 5]. Rawshani et al calculated HRs for the development of ASCVD according to the age of onset of type 1 diabetes using data from the Swedish National Diabetes Register. They included 27,195 individuals with type 1 diabetes and 135,178 matched control participants and showed that there is an inverse association between age at diagnosis and risk of ASCVD. Individuals with onset of type 1 diabetes before 10 years of age had an 11.4 times higher risk of ASCVD than matched control participants, and girls with disease onset before 10 years of age had a 13.2 times increased risk of ASCVD. The ASCVD risk was approximately three times higher in those with early-onset type 1 diabetes (<10 years of age) than in those with disease onset at age 26–30 years [1, 5]. After adjustment for duration of disease, these elevated risks remained. A recent study from the USA showed a peak incidence for type 1 diabetes at the age of 10 years (95% CI 8–11 years) [6]. Furthermore, the absolute risk of ASCVD in older individuals with type 1 diabetes, even those with relatively late onset of disease, is much higher than in matched control participants, with an age-adjusted incidence ratio between 2 and 5 [3]. The evidence on the deleterious impact of diabetes on ASCVD risk in children provides us with opportunities to search for treatment strategies to mitigate ASCVD risk early in the disease course.

## Pathophysiology of CVD in type 1 diabetes: role of lipoproteins

The mechanisms underlying the increased risk of atherosclerosis and ASCVD in type 1 diabetes are multifactorial and have only partially been elucidated, as outlined in a recent review [7], whereas the increased risk in familial hypercholesterolaemia (FH) is monocausal and the pathophysiology is much more straightforward [8]. Table 1 provides an outline of the similarities and differences between the pathophysiology of ASCVD in FH and type 1 diabetes. Nonetheless, glycaemic control has emerged as a key factor in ASCVD development in type 1 diabetes. Knowledge of the effects of glycaemic control was substantially increased by the DCCT, carried out in the 1980s and 1990s. Participants randomised to intensive treatment (HbA_1c_ levels in the normal range [<53 mmol/mol, <7%]) during the 6.5 year duration of the trial showed a significant reduction in microvascular complications compared with the conventional treatment group (HbA_1c_ levels in the high–normal range [<75 mmol/mol, <9%]). At the end of this trial, participants were enrolled in a 27 year follow-up study [11]. Despite the convergence of HbA_1c_ levels between the two groups, owing to the adoption of intensive therapy by the conventional treatment group, the development and progression of complications continued to be substantially less in the original intensive treatment group than in the conventional treatment group. There was a 57% lower risk of cardiovascular events in the intensive treatment group [12, 13]; this phenomenon was termed ‘metabolic memory’.

**Table 1:** Similarities and differences between ASCVD in FH and type 1 diabetes

Characteristic	FH	Type 1 diabetes
Onset	Birth	Peak incidence: early adolescence [9]
ASCVD risk	22-fold increased risk [10]	>10-fold increased risk [5]
Residual ASCVD risk on optimal therapy	Comparable to that in general population if treatment is started in early childhood	Increased
Cause of increased ASCVD risk	High LDL-cholesterol levels	Multifactorial; partly attributable to high LDL-cholesterol and oxidised LDL levels
Familial predisposition	Monogenetic dominant disease	Autoimmune disease, most likely with genetic vulnerability
Therapy: first line	Statins and others forms of lipid-lowering therapy, lifestyle changes	Tight glycaemic control (insulin)

**Fig. 1:**
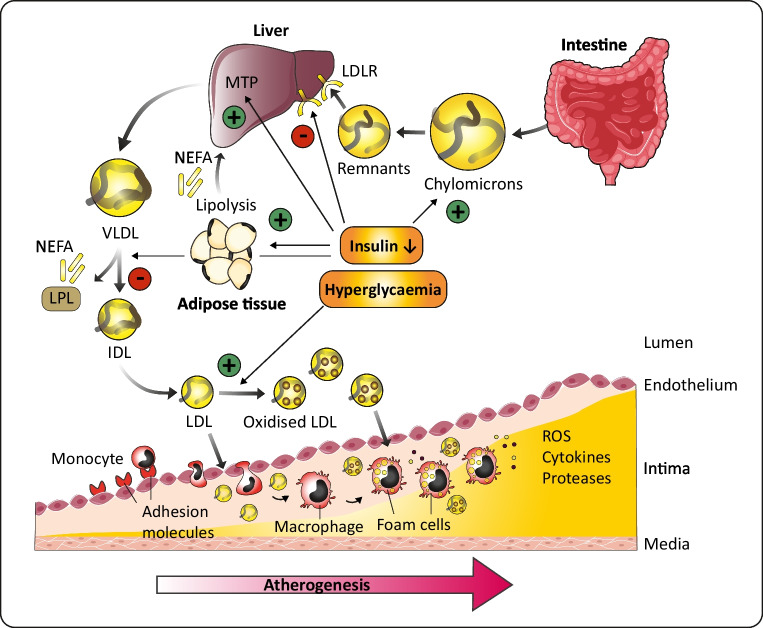
Atherogenic lipoproteins in individuals with type 1 diabetes with poor glycaemic control. Poor glycaemic control in type 1 diabetes drives the formation of atherogenic lipoproteins in several ways. Three of the key mechanisms are depicted here. First, decreased insulin levels lead to enhanced levels of chylomicrons. Decreased insulin levels promote chylomicron secretion by intestinal cells and inhibit LDLR-mediated uptake of chylomicron remnants by the liver. Second, decreased insulin levels lead to enhanced levels of circulating VLDL by fuelling adipose tissue lipolysis and thereby increasing the availability of NEFA for VLDL assembly. At the hepatic level, decreased insulin levels promote MTP-mediated VLDL assembly. VLDL catabolism is decreased by inhibition of lipoprotein lipase (LPL). Third, hyperglycaemia promotes the oxidation of LDL. Oxidised LDL drives the formation of immune complexes and is taken up by macrophages, which fuels the formation of macrophage foam cells, a hallmark of atherosclerotic plaque formation in the arterial intimal layer. IDL, intermediate-density lipoprotein; LDLR, LDL receptor; LPL, lipoprotein lipase; MTP, microsomal transfer protein; ROS, reactive oxygen species. This figure is available as a downloadable slide

It is known that poor glycaemic control leads to microalbuminuria and eventually nephropathy and hypertension, which are both associated with enhanced ASCVD risk in type 1 diabetes [7]. Moreover, poor glycaemic control coincides with dyslipidaemia. In the Coronary Artery Calcification Study in Type 1 Diabetes (CACTI), each 1% increase in HbA_1c_ was associated with a 0.1 mmol/l increase in LDL-cholesterol (LDL-c) [14]. The enhanced LDL-c levels in individuals with type 1 diabetes with poor glycaemic control are partly explained by the catabolic effects of insulin on LDL-c. Insulin enhances LDL receptor expression and activity, which lowers LDL-c levels [15, 16]. In fact, insulin affects lipoprotein metabolism at several levels, as illustrated in Fig. 1. Insulin increases lipoprotein lipase (LPL) activity, which hydrolises triglycerides in chylomicrons and VLDL and thereby promotes catabolism of these triglyceride-rich lipoproteins. Moreover, insulin suppresses the production of VLDL particles in the liver by inhibition of lipolysis and limiting the availability of NEFA as precursors for VLDL, and by inhibition of hepatic microsomal transfer protein (MTP), which is critical for hepatocyte VLDL assembly. Because of the pivotal role of insulin in lipoprotein metabolism, poor glycaemic control in type 1 diabetes is associated with high levels of atherogenic triglyceride-rich lipoproteins as well as cholesterol-rich LDL particles [15, 16].

In addition to these quantitative differences in lipoprotein levels, which are generally reversed by glycaemic control, type 1 diabetes is also associated with qualitative differences in lipoprotein species that are incompletely reversed by glycaemic control and which are also potentially atherogenic, as illustrated in Fig. 1. First, VLDL shows an increased cholesteryl ester/triglyceride ratio [15, 17]. The increased cholesterol content of VLDL may be explained by peripheral hyperinsulinaemia secondary to subcutaneous insulin administration, which promotes cholesteryl ester transfer protein (CETP) activity and hence the exchange of cholesteryl esters and triglycerides between VLDL and HDL. As a consequence, VLDL has an increased cholesterol content and HDL has an increased triglyceride content in type 1 diabetes. These differences can be reversed by intraperitoneal administration of insulin, which normalises CETP activity [18]. Second, hyperglycaemia in type 1 diabetes promotes the oxidation of LDL, partly mediated by hyperglycaemia-induced oxidative stress in endothelial cells [19]. The increased oxidation of LDL is associated with enhanced carotid intima–media thickness (cIMT) in young adults and adolescents with type 1 diabetes and poor glycaemic control [20] and promotes atherogenesis in different ways. Oxidised LDL (oxLDL) is taken up by macrophages in the subendothelial space and drives the formation of macrophage foam cells, one of the hallmarks of atherosclerosis development. Furthermore, circulating oxLDL evokes the formation of oxLDL antibodies, leading to the formation of oxLDL immune complexes. These oxLDL immune complexes are enhanced in individuals with type 1 diabetes and poor glycaemic control and have also been associated with increased ASCVD risk [20–22]. In summary, type 1 diabetes, and in particular poor glycaemic control, leads to quantitative and qualitative lipoprotein abnormalities that promote atherogenesis.

Recently, it has been shown that there is a clear association between increased LDL-c levels and a higher incidence of nephropathy and retinopathy in both children and adults [23]. This association remains present after statistical adjustment for glycaemic control, which could suggest that LDL-c (or more likely oxLDL or other unfavourable LDL subspecies) has a direct role in the pathogensis of these complications of type 1 diabetes. Dyslipidaemia therefore emerges as an important target for the prevention of ASCVD in type 1 diabetes.

## Lessons learned from familial hypercholesterolaemia

It is generally accepted that the retention of LDL-c within the arterial wall is the key initiating event in atherosclerosis [24]. Therefore, important lessons about the pathophysiology of atherosclerosis can be learned from FH. FH is a monogenetic, semi-dominant disorder affecting the LDL receptor, leading to decreased cellular uptake of LDL-c. This leads to (severely) elevated LDL-c levels, which are present from birth onwards. The disease exists in a heterozygous form (with a disease-causing mutation inherited from one parent) and a homozygous form (with a disease-causing mutation inherited from both parents). Heterozygous FH is the most common inherited metabolic disease, with an estimated prevalence of around 1 in 300 individuals in the general population [25, 26]. Homozygous FH is a rare disorder; prevalence has been estimated as 1 in 300,000 individuals.

Coronary atherosclerosis has been detected in men with heterozygous FH as young as 17 years of age and in women with heterozygous FH at age 25 years. In untreated individuals with FH, the mean age of the first cardiovascular event is 44 years [27]. As individuals with homozygous FH have mutations in both genes of the LDL receptor, they have no or hardly any functional LDL receptors and they therefore experience extremely high LDL-c levels from birth. As a consequence, untreated homozygous FH can lead to a myocardial infarction, which can be fatal, from the first decade of life [28, 29].

In countries where there is no screening for FH in healthy individuals, a large proportion of individuals with this disease remain undetected until the first cardiovascular event. However, since the introduction of statins in 1988, a very effective treatment for FH has become available. In the Netherlands, a nationwide screening programme for FH began in 1994 and therefore a large proportion of those with FH have been identified [30]. This enabled us to perform an RCT on the effects of early treatment with statins in young children with heterozygous FH [31]. Between 1997 and 1999, this double-blind trial enrolled 214 children with a mean age of 14±3.1 years from a single centre in Amsterdam. Participants were randomised to receive either pravastatin or placebo. After 24 months, it was shown that pravastatin reduced LDL-c levels by 25% compared with placebo, but also resulted in a significant regression of the cIMT [31]. In the open-label extension, all children were started on pravastatin. Twenty years after the original trial, the incidence of ASCVD in the (now young adult) participants was compared with that among their parents with FH for whom statin treatment became available at a much later age (mean age 32 years). Of the 214 participants in the original trial, information on cardiovascular events was obtained for 203 (95%). Only one had experienced a cardiovascular event (angina pectoris necessitating percutaneous coronary intervention); however, this participant discontinued statin use after the original trial and was a smoker. In the group of 156 parents with FH, 41 (26%) had a cardiovascular event before the age of 40 years (the youngest affected had a myocardial infarction at age 20 years), with 11 (7%) having a fatal infarction [32]. At the follow-up visit, 80% of the participants were still on lipid-lowering treatment (LLT) with good adherence. These data show that the process of atherosclerosis starts at an early age in individuals with high LDL-c levels, but also that this can be mitigated by the early start of LLT such as statins. In this group of individuals statins were found to be safe over a prolonged period and were well tolerated. There were no adverse effects on growth, sexual maturation, hormone levels, or liver or muscle tissue. The current treatment regimen for children with heterozygous FH is depicted in the textbox (‘Medical treatment for children with heterozygous FH in the Netherlands’).

## Initiation of statin treatment in children with diabetes

How does the evidence above translate to children with diabetes? Clearly, the pathophysiology of ASCVD in type 1 diabetes is multifactorial and the contribution of abnormal lipid metabolism is very complex, whereas in FH there is a strong correlation between high LDL-c levels and ASCVD. Nevertheless, the process of atherosclerosis can start early in life and this provides a strong rationale for aggressive treatment of risk factors in individuals at greater risk for ASCVD, such as children with type 1 diabetes, at an early age. In their scientific statement on ‘Cardiovascular risk reduction in high-risk pediatric patients’, the American Heart Association (AHA) classifies type 1 diabetes as a condition with a high risk for CVD, and its recommendations include stringent control of risk factors for ASCVD, including LDL-c levels. In the same statement the AHA classifies heterozygous FH as a moderate risk factor for ASCVD [34]. However, we disagree with this classification and rank heterozygous FH as a high-risk condition. Khera et al have clearly shown that the risk of ASCVD is underestimated if cholesterol levels are considered in isolation without considering heredity. The risk for ASCVD is increased sixfold for high LDL-c levels (>4.9 mmol/l) compared with normal LDL-c levels (<3.4 mmol/l), whereas LDL-c levels >4.9 mmol/l plus a pathogenic mutation for FH lead to a 22.3-fold increased risk of ASCVD [10]. Furthermore, in the AHA’s stratification, homozygous FH should be ranked ‘out of category’, because without immediate intensive treatment it may lead to ASCVD and death in the first decade of life [35].

As hyperglycaemia plays a pivotal role in the development of ASCVD in people with type 1 diabetes, optimisation of blood glucose levels should be the first priority. However, because an excess risk for ASCVD remains in individuals with well-regulated type 1 diabetes, other preventative measures should also be initiated. European and American guidelines recommend aggressive management of cardiovascular risk factors in individuals with diabetes, especially for those aged >40 years. Recent guidelines from the European Society of Cardiology (ESC) on the management of dyslipidaemias [36] recommend considering statin therapy in adults aged <40 years with type 1 or type 2 diabetes with evidence of target organ damage and/or LDL-c levels >2.6 mmol/l, as long as pregnancy is not planned. If lifestyle interventions have failed, the 2023 ADA guidelines recommend ‘considering’ the addition of a statin in youth aged >10 years with type 1 diabetes who continue to have total cholesterol levels >4.1 mmol/l or LDL-c levels >3.4 mmol/l plus another cardiovascular risk factor [37]. Recent guidelines from the International Society for Pediatric and Adolescent Diabetes (ISPAD) also advise considering statins after the age of 10 years if, despite lifestyle changes, LDL-c levels remain >3.4 mmol/l. In the ISPAD guidelines the presence of additional ASCVD risk factors are not mentioned [38]. These recommendations are largely based on data extrapolated from adult studies and expert opinion. As FH has a high prevalence, it should also be considered whether a child might be affected by both type 1 diabetes and FH, especially if there is a family history of early-onset ASCVD. If a child is affected by both type 1 diabetes and FH, we recommend starting statin treatment according to the guidelines shown in the textbox (‘Medical treatment for children with heterozygous FH in the Netherlands’).

Although a few RCTs have been carried out in children from age 10 years with type 1 diabetes to assess the effect of early statin use on surrogate markers for ASCVD [39–41], no trials have been carried out on the development of ASCVD over the longer term. Clearly these trials are hampered by the need for a follow-up period of many years. Because long-term data are not yet available, the use of LLT in children with type 1 diabetes for the prevention of ASCVD remains controversial. The Pediatric Atorvastatin in Diabetes Trial (PADIT) [40] was a small RCT with a crossover design that investigated the effects of 12 weeks’ treatment with atorvastatin compared with placebo in 51 participants (*n*=25 male, age 10–21 years) with type 1 diabetes. As expected, the use of atorvastatin resulted in a significant decrease in LDL-c (by 0.75±0.51 mmol/l), with no aspartate aminotransferase or alanine aminotransferase elevations more than twice the upper limit of normal or changes in serum creatine kinase observed. Although there were no significant effects on the primary endpoint (arterial stiffness during crossover), the trial did provide some evidence that the use of atorvastatin might be associated with reduced arterial stiffness, although the difference reported was not significant. However, it should be noted that the intervention period of 12 weeks was probably too short to show significant results. In another small RCT by Canas et al, in which 42 participants with type 1 diabetes were randomised to placebo or atorvastatin treatment for 6 months, it was again shown that LDL-c levels were effectively decreased by statin use [39]. However, in this trial the decrease in LDL-c levels was mostly caused by a reduction in levels of the larger, less atherogenic LDL particles. Statin safety was found to be excellent in this trial, with one participant experiencing elevation of creatine kinase levels, which normalised after statin discontinuation [39]. No effects on glycaemic control were noted; information on the effects of surrogate markers of ASCVD, such as cIMT measurements, was lacking; and the duration of treatment was short. The largest RCT on statin use in children with type 1 diabetes to date was performed by Loredana Marcovecchio et al [42]. In this relatively large trial, 443 adolescents were randomly assigned to placebo, an ACE inhibitor or a statin with the use of a 2×2 factorial design. The median duaration of follow-up was 2.6 years. The trial did not show significant effects of statins on cIMT, the primary vascular marker of the trial; however, it did show that the use of statins significantly reduced lipid levels in children with type 1 diabetes, with no safety issues.

Clearly, there is a need for long-term RCTs of LLT in children with type 1 diabetes aged ≥10 years, or even younger children in the case of early-onset of disease, to provide firm evidence on the benefits of LLT. Validated surrogate markers for ASCVD, such as cIMT and carotid–femoral pulse wave velocity, should be used as primary endpoints and sample sizes should be large enough to enable long-term follow-up. Target LDL-c levels should also be given special consideration. In individuals with good glycaemic control and LDL-c levels within the target range, abnormal LDL-c subclasses can persist [16]. More knowledge about the clinical effects of these abnormal subclasses will provide insight into how aggressively lipid levels should be managed in children and young adults with type 1 diabetes. It remains unclear if children with type 1 diabetes or children with FH need to be set the lowest LDL-c targets, as FH starts at birth whereas type 1 diabetes develops later in life. To draw firm conclusions, many more children with type 1 diabetes than currently should be treated with statins. Undertreatment also appears to be an issue in adults, for whom more evidence exists for the benefits of tight control of dyslipidaemia in diabetes [43].

In recent years, several new types of drugs that regulate LDL-c levels, such as bempedoic acid and proprotein convertase subtilisin/kexin type 9 (PCSK9) inhibitors [44–46], have become available. In the next few years, the availability of further RCT data on the use of these drugs in children and young adults with FH might also provide valuable information for children with type 1 diabetes.

## Considerations when starting statin therapy

In our 30 years’ experience of statin use in children with FH we have not observed any serious side effects, such as episodes of rhabdomyolysis. Elevations of creatine kinase have been noted only sporadically, specifically in families who turned out to be statin-intolerant. A few meta-analyses and systematic reviews of trials in which statins were administered to children for up to 2 years have confirmed that statins have very few adverse effects or side effects, and that the most common side effects experienced by children are headache, abdominal complaints and myalgia [47]. These side effects are transient and there are very few differences according to the type and dose of statin. How to accurately diagnose and manage true statin intolerance is described in detail elsewhere [47]. Regular (e.g. every 3–12 months) blood tests are advisable when children are on statin therapy, which can be combined with diabetes check-ups.

Statin use has been associated with disease progression in type 2 diabetes [48] but this is most likely due to an increase in peripheral insulin resistance, which is the hallmark of type 2 diabetes but less of an issue in type 1 diabetes. Nevertheless, it should be noted that, with the current obesity epidemic, children and young adults may experience a combination of type 1 diabetes with features of insulin resistence and type 2 diabetes (or other forms of insulin resistence) at the same time.

Another important issue to consider is medication adherence. FH patients usually show good adherence to statin therapy, as we found in our RCT [31] and follow-up study [49]. However, for most children with FH this is their only medical issue, whereas children with type 1 diabetes need to administer/regulate their insulin and perhaps also take other oral medication such as ACE inhibitors, which is a very demanding task during childhood or adolescence. This issue was addressed in the Adolescent Type 1 Diabetes Cardio-Renal Intervention Trial (AdDIT) [41], which found an overall adherence rate for statins of around 80%, which is comparable to our results in FH patients. Nevertheless, physicians should inform patients about why statins are prescribed and why it is important to adhere to the treatment.

One final aspect of initiating statin treatment in adolescents is the contraindication to statins during pregnancy. Cholesterol plays an important role in embryogenesis and in animal studies high doses of statins have shown teratogenic effects [50]. There are not enough data from human studies to conclude that lower doses of statins during pregnancy are safe. Therefore, pregnant women and sexually active females of reproductive age who are trying to conceive should be advised to use contraceptives and temporarily discontinue statin use.

## Conclusion

Thirty years of experience of treating children with FH has shown that statins are safe, well tolerated and effective. It has been shown that early (from 8–10 years of age) initiation of statin treatment dramatically reduces the incidence of ASCVD and mortality in young adults with FH. Young adults who have been diagnosed with type 1 diabetes in childhood still have a very high risk for early-onset ASCVD, despite the improvements in care for people with diabetes that have been made in the last few decades. Although the pathogenesis of ASCVD in type 1 diabetes is multifactorial, and long-term trials of LLT in children, including imaging, are lacking, there is a strong rationale for early and aggressive ASCVD risk management in children with type 1 diabetes, with an increasing role for statins. Newer types of LLT should be a topic of intensive research.

### Supplementary Information

Below is the link to the electronic supplementary material.

Supplementary file1 (PPTX 327 KB)

## Abbreviations

ASCVD: Atherosclerotic cardiovascular disease
CACTI: Coronary Artery Calcification Study in Type 1 Diabetes
CETP: Cholesteryl ester transfer protein
cIMT: Carotid intima–media thickness
FH: Familial Hypercholesterolaemia
LDL-c: LDL-cholesterol
LLT: Lipid-lowering treatment
LPL: Lipoprotein lipase
MTP: Microsomal transfer protein
oxLDL: Oxidised LDL

## References

[1] Rawshani A, Rawshani A, Franzen S (2017). Mortality and cardiovascular disease in type 1 and type 2 diabetes. N Engl J Med.

[2] Lind M, Svensson AM, Kosiborod M (2014). Glycemic control and excess mortality in type 1 diabetes. N Engl J Med.

[3] Livingstone SJ, Looker HC, Hothersall EJ (2012). Risk of cardiovascular disease and total mortality in adults with type 1 diabetes: Scottish registry linkage study. PLoS Med.

[4] Secrest AM, Becker DJ, Kelsey SF, LaPorte RE, Orchard TJ (2010). All-cause mortality trends in a large population-based cohort with long-standing childhood-onset type 1 diabetes: the Allegheny County type 1 diabetes registry. Diabetes Care.

[5] Rawshani A, Sattar N, Franzen S (2018). Excess mortality and cardiovascular disease in young adults with type 1 diabetes in relation to age at onset: a nationwide, register-based cohort study. Lancet.

[6] Wagenknecht LE, Lawrence JM, Isom S (2023). Trends in incidence of youth-onset type 1 and type 2 diabetes in the USA, 2002–18: results from the population-based SEARCH for Diabetes in Youth study. Lancet Diabetes Endocrinol.

[7] Verges B (2020). Cardiovascular disease in type 1 diabetes: a review of epidemiological data and underlying mechanisms. Diabetes Metab.

[8] Martin AC, Gidding SS, Wiegman A, Watts GF (2017). Knowns and unknowns in the care of pediatric familial hypercholesterolemia. J Lipid Res.

[9] Diaz-Valencia PA, Bougneres P, Valleron AJ (2015). Global epidemiology of type 1 diabetes in young adults and adults: a systematic review. BMC Public Health.

[10] Khera AV, Won HH, Peloso GM (2016). Diagnostic yield and clinical utility of sequencing familial hypercholesterolemia genes in patients with severe hypercholesterolemia. J Am Coll Cardiol.

[11] Diabetes Control and Complications Trial Research Group (1993). The effect of intensive treatment of diabetes on the development and progression of long-term complications in insulin-dependent diabetes mellitus. N Engl J Med.

[12] Nathan DM, Cleary PA, Backlund JY (2005). Intensive diabetes treatment and cardiovascular disease in patients with type 1 diabetes. N Engl J Med.

[13] Nathan DM (2021). Realising the long-term promise of insulin therapy: the DCCT/EDIC study. Diabetologia.

[14] Maahs DM, Ogden LG, Dabelea D (2010). Association of glycaemia with lipids in adults with type 1 diabetes: modification by dyslipidaemia medication. Diabetologia.

[15] Verges B (2009). Lipid disorders in type 1 diabetes. Diabetes Metab.

[16] Verges B (2020). Dyslipidemia in type 1 diabetes: amasked danger. Trends Endocrinol Metab.

[17] Caixas A, Perez A, Payes A (1998). Effects of a short-acting insulin analog (Insulin Lispro) versus regular insulin on lipid metabolism in insulin-dependent diabetes mellitus. Metabolism.

[18] Bagdade JD, Dunn FL, Eckel RH, Ritter MC (1994). Intraperitoneal insulin therapy corrects abnormalities in cholesteryl ester transfer and lipoprotein lipase activities in insulin-dependent diabetes mellitus. Arterioscler Thromb.

[19] Tanaka J, Qiang L, Banks AS (2009). Foxo1 links hyperglycemia to LDL oxidation and endothelial nitric oxide synthase dysfunction in vascular endothelial cells. Diabetes.

[20] Hunt KJ, Baker N, Cleary P (2013). Oxidized LDL and AGE-LDL in circulating immune complexes strongly predict progression of carotid artery IMT in type 1 diabetes. Atherosclerosis.

[21] Virella G, Lopes-Virella MF (2008). Atherogenesis and the humoral immune response to modified lipoproteins. Atherosclerosis.

[22] Engelen SE, Robinson AJB, Zurke YX, Monaco C (2022). Therapeutic strategies targeting inflammation and immunity in atherosclerosis: how to proceed?. Nat Rev Cardiol.

[23] Rathsman B, Haas J, Persson M (2021). LDL cholesterol level as a risk factor for retinopathy and nephropathy in children and adults with type 1 diabetes mellitus: a nationwide cohort study. J Intern Med.

[24] Kaplan M, Aviram M (1999). Oxidized low density lipoprotein: atherogenic and proinflammatory characteristics during macrophage foam cell formation. An inhibitory role for nutritional antioxidants and serum paraoxonase. Clin Chem Lab Med.

[25] Beheshti SO, Madsen CM, Varbo A, Nordestgaard BG (2020). Worldwide prevalence of familial hypercholesterolemia: meta-analyses of 11 million subjects. J Am Coll Cardiol.

[26] Hu P, Dharmayat KI, Stevens CAT (2020). Prevalence of familial hypercholesterolemia among the general population and patients with atherosclerotic cardiovascular disease: a systematic review and meta-analysis. Circulation.

[27] Krogh HW, Mundal L, Holven KB, Retterstol K (2016). Patients with familial hypercholesterolaemia are characterized by presence of cardiovascular disease at the time of death. Eur Heart J.

[28] Widhalm K, Benke IM, Fritz M (2017). Homozygous familial hypercholesterolemia: summarized case reports. Atherosclerosis.

[29] Keller C (2009). LDL-apheresis in homozygous LDL-receptor-defective familial hypercholesterolemia: the Munich experience. Atheroscler Suppl.

[30] Louter L, Defesche J, Roeters van Lennep J (2017). Cascade screening for familial hypercholesterolemia: practical consequences. Atheroscler Suppl.

[31] Wiegman A, Hutten BA, de Groot E (2004). Efficacy and safety of statin therapy in children with familial hypercholesterolemia: a randomized controlled trial. JAMA.

[32] Luirink IK, Wiegman A, Kusters DM (2019). 20-year follow-up of statins in children with familial hypercholesterolemia. N Engl J Med.

[33] Climent E, Benaiges D, Pedro-Botet J (2021). Hydrophilic or lipophilic statins?. Front Cardiovasc Med.

[34] de Ferranti SD, Steinberger J, Ameduri R (2019). Cardiovascular risk reduction in high-risk pediatric patients: a scientific statement from the American Heart Association. Circulation.

[35] Galiano M, Hammersen J, Sauerstein K (2020). Homozygous familial hypercholesterolemia with severe involvement of the aortic valve: a sibling-controlled case study on the efficacy of lipoprotein apheresis. J Clin Apharesis.

[36] Mach F, Baigent C, Catapano AL (2020). 2019 ESC/EAS Guidelines for the management of dyslipidaemias: lipid modification to reduce cardiovascular risk. Eur Heart J.

[37] ElSayed NA, Aleppo G, Aroda VR (2023). 14. Children and adolescents: Standards of Care in Diabetes-2023. Diabetes Care.

[38] Bjornstad P, Dart A, Donaghue KC (2022). ISPAD Clinical Practice Consensus Guidelines 2022: microvascular and macrovascular complications in children and adolescents with diabetes. Pediatr Diabetes.

[39] Canas JA, Ross JL, Taboada MV (2015). A randomized, double blind, placebo-controlled pilot trial of the safety and efficacy of atorvastatin in children with elevated low-density lipoprotein cholesterol (LDL-C) and type 1 diabetes. Pediatr Diabetes.

[40] Haller MJ, Stein JM, Shuster JJ (2009). Pediatric Atorvastatin in Diabetes Trial (PADIT): a pilot study to determine the effect of atorvastatin on arterial stiffness and endothelial function in children with type 1 diabetes mellitus. J Pediatr Endocrinol Metab.

[41] Niechcial E, Acerini CL, Chiesa ST (2020). Medication adherence during adjunct therapy with statins and ACE inhibitors in adolescents with type 1 diabetes. Diabetes Care.

[42] Loredana Marcovecchio M, Chiesa ST, Bond S (2017). ACE inhibitors and statins in adolescents with type 1 diabetes. N Engl J Med.

[43] Zgibor JC, Wilson RR, Orchard TJ (2005). Has control of hypercholesterolemia and hypertension in type 1 diabetes improved over time?. Diabetes Care.

[44] Santos RD, Ruzza A, Hovingh GK (2022). Paediatric patients with heterozygous familial hypercholesterolaemia treated with evolocumab for 80 weeks (HAUSER-OLE): a single-arm, multicentre, open-label extension of HAUSER-RCT. Lancet Diabetes Endocrinol.

[45] Santos RD, Ruzza A, Hovingh GK (2020). Evolocumab in pediatric heterozygous familial hypercholesterolemia. N Engl J Med.

[46] Ray KK, Bays HE, Catapano AL (2019). Safety and efficacy of bempedoic acid to reduce LDL cholesterol. N Engl J Med.

[47] Alonso R, Cuevas A, Cafferata A (2019). Diagnosis and management of statin intolerance. J Atheroscler Thromb.

[48] Mansi IA, Chansard M, Lingvay I, Zhang S, Halm EA, Alvarez CA (2021). Association of statin therapy initiation with diabetes progression: a retrospective matched-cohort study. JAMA Intern Med.

[49] Braamskamp MJ, Kusters DM, Avis HJ (2015). Long-term statin treatment in children with familial hypercholesterolemia: more insight into tolerability and adherence. Paediatr Drugs.

[50] Jyoti S, Tandon S (2015). Genetic basis for developmental toxicity due to statin intake using embryonic stem cell differentiation model. Hum Exp Toxicol.

