# Supplementary material for: Dyslipidaemia as a target for atherosclerotic cardiovascular disease prevention in children with type 1 diabetes: lessons learned from familial hypercholesterolaemia
Source: Diabetologia. 2023 Nov 30;67(1):19–26. doi: 10.1007/s00125-023-06041-z (PMC10709243; doi:10.1007/s00125-023-06041-z)
Supplement: Supplementary file 1 — Supplementary file1 (PPTX 327 KB) [file 125_2023_6041_MOESM1_ESM.pptx]

## Slide 1
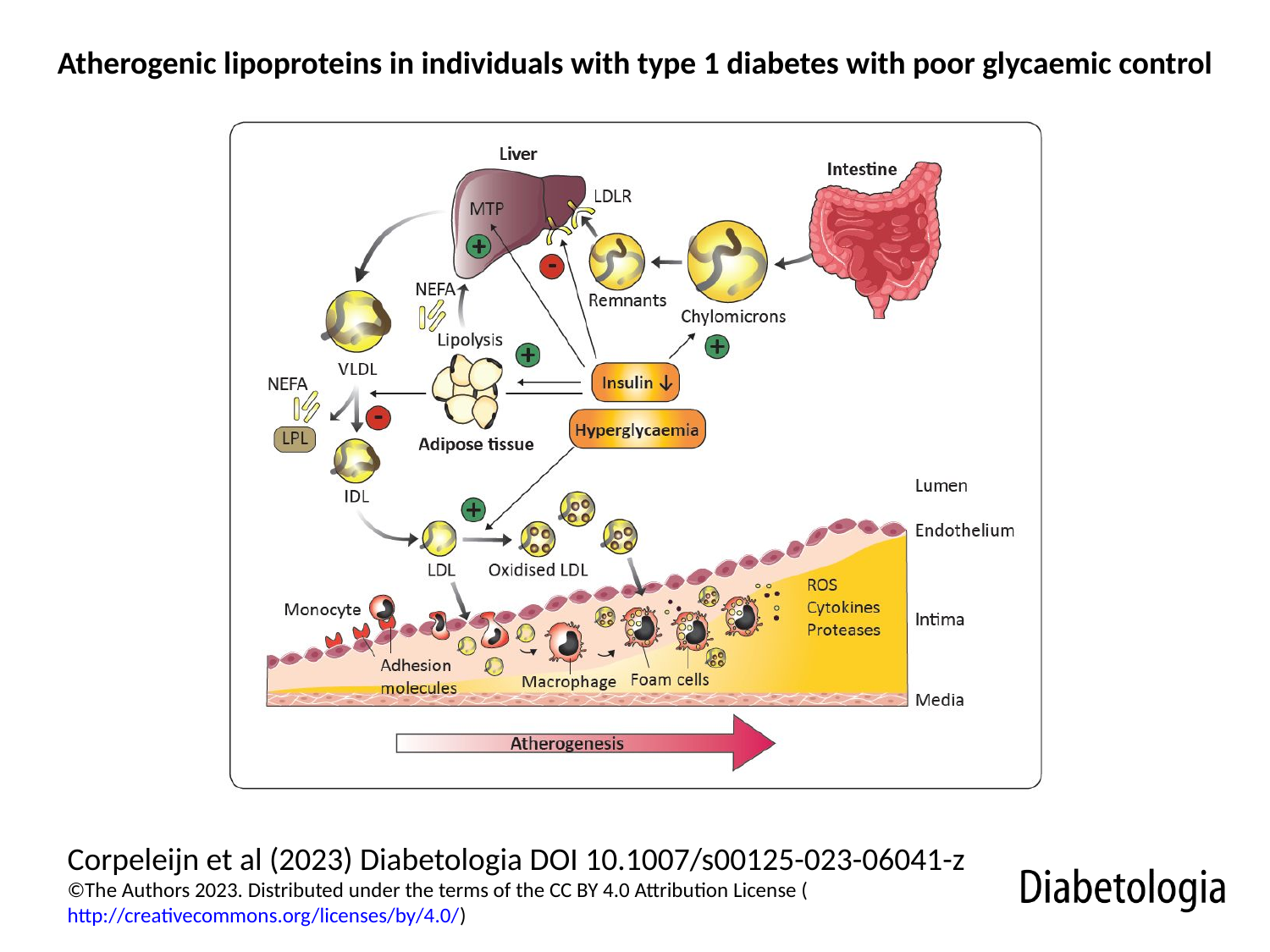

Atherogenic lipoproteins in individuals with type 1 diabetes with poor glycaemic control
Corpeleijn et al (2023) Diabetologia DOI 10.1007/s00125-023-06041-z
©The Authors 2023. Distributed under the terms of the CC BY 4.0 Attribution License (http://creativecommons.org/licenses/by/4.0/)
